# Etiology of panurethral strictures in a low socioeconomic status population

**DOI:** 10.1007/s11255-024-04328-7

**Published:** 2024-12-17

**Authors:** Kunj Jain, Radhika Patel, Aleksandar Popovic, Meher Pandher, Amjad Alwaal

**Affiliations:** Department of Surgery, Division of Urology, Rutgers New Jersey Medical School, 185 South Orange Avenue, Newark, NJ 07103 USA

**Keywords:** Panurethral strictures, Lichen sclerosis

## Abstract

**Introduction:**

Panurethral strictures represent the most severe form within the anterior urethral stricture spectrum, requiring more technically complex repairs and resulting in poorer outcomes compared to localized anterior urethral strictures (penile or bulbar). This abstract aims to describe the distinct characteristics of patients with panurethral strictures in a low socioeconomic status population.

**Methods:**

Patients presenting with localized anterior (penile or bulbar) or panurethral strictures at University Hospital in Newark, NJ, between 2021 and 2023 were retrospectively identified. Data were extracted from electronic medical records and analyzed statistically using IBM SPSS Software.

**Results:**

Among the patients, 33 had localized anterior urethral strictures, and 22 had panurethral stricture disease. Hispanic and African American patients accounted for the majority of stricture cases (63.6%), including 59% of the panurethral stricture cohort. The only statistically significant factor contributing to panurethral disease was lichen sclerosis (*p* < 0.05). Patients with panurethral strictures had a higher incidence of inflammatory and systemic diseases such as STDs, recurrent UTIs, diabetes, and hypertension, while those with localized anterior urethral strictures showed a higher incidence of iatrogenic factors, including prior catheterizations and transurethral surgeries. However, these factors did not reach a statistical significance. Hypospadias repair was observed in 6% of localized anterior urethral stricture cases, compared to 13.6% of panurethral stricture cases.

**Conclusion:**

While iatrogenic causes remain the predominant contributors, inflammatory and systemic conditions, particularly lichen sclerosis, significantly influence the development of panurethral strictures. Early surgical intervention and better management of systemic diseases may prevent the progression of localized anterior urethral strictures to panurethral disease, but further studies utilizing larger number of patients may shed light on the significance of these systemic factors.

## Introduction

Urethral stricture disease is a relatively common condition, with a prevalence of 0.6% among men in the U.S. [1]. In industrialized countries, the primary causes of urethral strictures are iatrogenic, stemming from procedures such as catheterization, cystoscopy, transurethral surgeries, or prior hypospadias repair [2]. Urethral stricture disease presents as a spectrum, from localized anterior strictures—affecting areas like the penile or bulbar urethra—to panurethral strictures, which involve the entire anterior urethra and are significantly more complex and challenging to manage.

Panurethral strictures represent a severe and technically demanding form of the disease, often associated with poorer surgical outcomes and requiring more intricate management strategies than localized anterior strictures. In addition to the need for more complex surgical interventions, panurethral strictures are often influenced by patient-specific factors, which can contribute to their development and progression. These factors include the underlying cause of the stricture, prior urological procedures, and the availability of autologous tissue for grafts or flaps [3]. Furthermore, patient comorbidities such as diabetes, inflammatory conditions, or other systemic diseases can exacerbate the complexity of managing panurethral strictures. Recognizing these characteristics is essential for developing tailored treatment strategies that improve patient outcomes.

This study seeks to identify the distinct patient characteristics associated with panurethral stricture disease compared to localized anterior strictures. By analyzing data from patients treated at a tertiary referral center serving a low socioeconomic population, we aim to uncover demographic and clinical factors that may influence the development of panurethral strictures. These findings could help inform earlier intervention strategies and refine management protocols, ultimately contributing to advancements in surgical techniques and improving outcomes for patients facing this challenging condition.

## Methods

### Study design and setting

This study employed a retrospective cohort design to evaluate patients diagnosed with urethral strictures who underwent surgical intervention at University Hospital in Newark, NJ, between 2021 and 2023. The primary focus was to assess patient demographics, clinical characteristics, and surgical outcomes across different urethral stricture types, specifically localized anterior (penile or bulbar) and panurethral strictures.

### Patient identification and selection

Patients were identified through a comprehensive review of the hospital’s electronic medical records (EMRs). The inclusion criteria were: 1) Diagnosis of Urethral Stricture, where patients had anterior (penile or bulbar) or panurethral strictures confirmed through clinical evaluation and imaging studies, and 2) Surgical Intervention, including patients who underwent surgical procedures such as various types of urethroplasty for stricture management. Exclusion criteria included: Incomplete Records, referring to patients with missing or insufficient data on surgical history or clinical outcomes.

### Data collection

Data were extracted from EMRs and included variables such as demographic information (age, sex, race/ethnicity), medical history (smoking status, diabetes, hypertension, body mass index [BMI], prior catheterization, history of hypospadias repair, and previous transurethral surgeries), stricture characteristics (location of the stricture, type of surgery performed such as buccal mucosa or anastomotic urethroplasty), surgical history (number and types of previous surgeries), and outcomes (post-surgical complications, stricture recurrence, recurrent urinary tract infections [UTIs], and history of lichen sclerosis or sexually transmitted diseases [STDs]).

### Data analysis

The collected data were analyzed using IBM SPSS Statistics Software (version 28). Descriptive statistics were employed to calculate means, medians, and standard deviations for continuous variables like age and BMI, and frequencies and percentages for categorical variables such as smoking history and diabetes status. Independent t tests were used for continuous variables and chi-square tests for categorical variables to compare differences between patients with anterior and panurethral strictures. Regression analysis was performed to assess the impact of various factors on surgical outcomes, including stricture recurrence and post-surgical complications, based on clinical relevance and preliminary analysis. A *p*-value of less than 0.05 was considered statistically significant for all analyses.

### Ethical considerations

The study adhered to ethical standards, with patient confidentiality maintained through de-identification of data during analysis. Institutional Review Board (IRB) approval was obtained from University Hospital in Newark, NJ, ensuring compliance with ethical guidelines and the protection of patient rights.

## Results

The study analyzed patient demographics for urethral strictures, categorizing patients based on the location of the stricture into anterior (*n* = 33) and panurethral (*n* = 22) groups. Key variables examined included patient demographics, medical history, and surgical history.

Patients with anterior urethral stricture (penile or bulbar) had a mean age of 46.8 years, while those with panurethral strictures had a higher mean age of 56.9 years. In the anterior stricture group, the racial distribution was Black (30.3%), Hispanic (33.3%), Caucasian (15.2%), and Asian (9.1%). For panurethral strictures, the racial breakdown was Caucasian (22.7%), Black (18.2%), Hispanic (45.5%), and Asian (13.6%). The prevalence of diabetes was higher in the panurethral group (22.7%) compared to the anterior group (9.1%). Hypertension was also more common in the panurethral group (40.9%) compared to the anterior group (30.3%). Significant differences were found in several baseline characteristics, including age, smoking history, BMI, and comorbid conditions (Table 1).

**Table 1:** Characteristics of stricture patients

Category	Characteristic	Anterior stricture (%) (*N* = 33)	Panurethral stricture (%) (*N* = 22)	*P*-value
Baseline characteristics	Age	46.8	56.9	0.186
	Smoking history	48.5	45.5	0.825
	Diabetes	9.1	22.7	0.159
	HTN	30.3	40.9	0.418
	BMI	27.9	31.7	0.055
Urologic history	Prior catheterization	72.7	59.1	0.291
	Cystoscopy	72.7	81.8	0.446
	Hypospadias repair	6.1	13.6	0.3383
	Transurethral surgery	45.5	45.5	1.00
Inflammatory history	Lichen sclerosis	3.0	27.3	0.0082*
	STD	6.1	9.1	0.672
	recurrent UTI	24.2	27.3	0.801

*HTN* hypertension, *STD* sexually transmitted disease, *UTI* urinary tract infection. *p < 0.05

Although there was no significant difference in overall urologic history between groups, prior catheterization was more frequently seen in patients with isolated anterior urethral strictures (72.7% vs. 59.1%). Additionally, there was an increased rate of prior hypospadias repair in panurethral stricture patients (13.6% vs. 6.1%). Both groups had an equal history of transurethral surgery, with 45.5% in each group (See Table 1).

Inflammatory etiologies were more commonly observed in panurethral stricture patients. Lichen sclerosis was present in 27.3% of panurethral cases, compared to 3.0% of isolated anterior cases. Panurethral stricture patients also had a higher incidence of recurrent urinary tract infections (UTIs) and sexually transmitted diseases (STDs), with rates of 9.1% vs. 6.1% for UTIs and 27.3% vs. 24.2% for STDs, respectively (See Table 1).

## Discussion

The etiology of urethral strictures has evolved considerably over time. In the 1960s, the most common cause was urethritis related to STDs, while in the 1980s, iatrogenic causes predominated. Our study found that only 7.3% of patients had urethral strictures and STDs, compared to a prior study by Lumen et al. which demonstrated a rate of 3.7% [2]. The studied population may have increased rates of STD-related strictures due to decreased condom usage in this low socioeconomic status population [4].

In Western countries, lichen sclerosis is the most common inflammatory cause of strictures. Although rare, with a prevalence of 0.1–0.3%, lichen sclerosis was responsible for 27% of panurethral strictures in our study, compared to just 3% in anterior strictures [5]. Other studies have shown that lichen sclerosis can result in strictures in up to 50% of patients [6]. Lichen sclerosis is an inflammatory process that progressively affects the prepuce, glans, and meatus. As the disease progresses further, there is urethral involvement starting from the penile urethra and progressing to panurethral involvement [5, 7]. Since the risk of panurethral development is high, meatal or isolated distal anterior urethral strictures should be treated promptly in order to prevent progression to panurethral strictures.

Prior catheterization and cystoscopies are well-established risk factors for urethral strictures and was shown in up to 80% of patients in our study population. 15–25% of hospitalized patients receive indwelling urinary catheters and these rates can even exceed 90% in intensive care units [2, 8]. The appropriateness of these catheters varies with some studies suggesting that 21–31% of the catheterized patients do not meet the criteria for use [9]. A recent study shows that 5.3 urethral injuries per 1000 catheterizations occurred in men [10]. Traumatic urethral catheterization results in increased need for cystoscopic intervention, urinary tract infections, and prolonged catheterization, all of which can lead to urethral strictures.

In our study, iatrogenic causes, especially transurethral surgery, were implicated in nearly 50% of cases, which is in accordance with other major series which suggest rates between 30 and 80% [11–14]. Several causes of stricture after transurethral surgery have been proposed, including traumatic insertion, mucosal injury, and instrument friction due to acute penoscrotal angle [15]. However, the true cause of stricture after surgery is undetermined, in our study, it resulted in both anterior and panurethral strictures equally. In addition, patients undergoing transurethral surgery are catheterized in the post-operative period, which compounds the rate of urethral strictures.

Idiopathic strictures are extremely uncommon as observed by multiple studies [11, 12, 14]. Our study had one patient, a 60-year-old Asian male, with an anterior stricture without any identifiable causes.

Smoking was not associated with increased risk of panurethral disease. Patient co-morbidities such as diabetes, and hypertension were associated with increased risk of panurethral strictures but was not statistically significant. These co-morbidities can compromise vasculature and result in urethral strictures due to ischemia. Panurethral stricture patients had a higher BMI and while no prior studies have shown a correlation between BMI and urethral strictures, multiple studies have shown that elevated BMI increase the risk of stricture recurrence after urethroplasty [16, 17]. These factors did not, however, reach statistical significance.

This study has several limitations, including its single-institutional design and small sample size at a tertiary referral center that were referred for urethroplasty. As such, the results may be biased as stricture etiology may differ in patients treated at smaller centers, where less invasive treatments such as internal urethrotomy or dilation may be more common. However, this study provides valuable insights into the etiology of panurethral strictures, particularly in an underserved, lower socioeconomic patient population. Future studies are needed with a larger multi-center study to confirm these results and remove biases that are inherent in this study. Our study may not have been powered enough to confirm systemic factor influence on the development of panurethral stricture disease.

While iatrogenic causes continue to be the primary contributors, lichen sclerosis significantly influences the development of panurethral strictures. Timely surgical intervention and improved management of the disease and comorbidities could halt the progression of localized anterior strictures to panurethral disease, thereby enhancing long-term patient outcomes. Future studies are needed to confirm if systemic factors are important in the development of panurethral stricture disease.

## Data Availability

No datasets were generated or analysed during the current study.
